# Innovative therapeutic approach to a rare severe amivantamab-related scalp toxicity: A case report

**DOI:** 10.1016/j.jdcr.2026.08.013

**Published:** 2026-08-17

**Authors:** Sara Morakabian, Mohammed Y. Youssef, Yaseen Ahmad, Abhishek Sinha, Marc Demyun

**Affiliations:** a The University of Texas Medical Branch, John Sealy School of Medicine, Galveston, Texas; b Internal Medicine Department, Hunt Regional Medical Center, Greenville, Texas; c Family Medicine Department, Texoma Medical Center, Sherman, Texas; d Countess of Chester NHS Foundation Trust, Chester, UK

**Keywords:** amivantamab, amniotic allograft, EGFR inhibitor, erosive pustular dermatosis, non-small cell lung cancer, oncodermatology, scalp toxicity, topical propranolol, wound healing

## Background

Amivantamab is a bispecific monoclonal antibody targeting both the epidermal growth factor receptor (EGFR) and mesenchymal-epithelial transition (MET) receptor. It was first approved by the U.S. Food and Drug Administration in May 2021 for the treatment of patients with locally advanced or metastatic non-small cell lung cancer harboring EGFR exon 20 insertion mutations, a population with limited targeted therapeutic options.^1^ Dermatologic toxicities are among the most common adverse effects and include acneiform rash, pruritus, paronychia, and scalp involvement.^2^ A recent multicenter study of 135 patients reported scalp ulcerative lesions in 31.1% of patients treated with amivantamab, representing the third most common dermatologic adverse event, with 26.7% experiencing grade ≥3 toxicities.^3^ Current management guidelines recommend multidisciplinary monitoring and early intervention to mitigate treatment-related cutaneous toxicities.^4^

Erosive pustular dermatosis of the scalp (EPDS) is a rare, chronic inflammatory disorder that typically affects older individuals with actinic damage, scarring, or prior local trauma.^5^ It is characterized by sterile pustules and crusted erosions of the scalp and is frequently mistaken for infection, cutaneous malignancy, or pyoderma gangrenosum; histopathologic findings are generally nonspecific.^6^ Reported triggers include local trauma (eg, surgery or skin grafting), photodynamic therapy, and medications. First-line treatment typically consists of high-potency topical corticosteroids, topical tacrolimus, or systemic retinoids, although relapses are common.^7^ Within oncology, EPDS has been reported rarely in association with EGFR inhibitors such as gefitinib. Its pathogenesis in this setting is thought to involve disruption of epidermal homeostasis and impaired wound repair, processes that may be further exacerbated by dual EGFR/MET inhibition.^8^ Although a growing body of literature has described amivantamab-associated scalp toxicity, severe ulcerative presentations consistent with EPDS remain uncommon, and the optimal management of refractory cases has yet to be established.

## Case presentation

A Caucasian woman in her 70s with stage IVB non-small cell lung cancer and concurrent multiple myeloma was found to harbor an EGFR exon 20 insertion mutation. She had previously received platinum-based chemotherapy plus immunotherapy; however, disease progression prompted initiation of amivantamab as salvage therapy.

Within 4 weeks of starting amivantamab, she developed a diffuse papulopustular eruption involving the scalp, trunk, and extremities (Common Terminology Criteria for Adverse Events grade 2-3). The scalp lesions consisted of folliculocentric papules and pustules with overlying crusting distributed across the vertex and bilateral parietal scalp, accompanied by erythema, tenderness, and significant pruritus. Owing to these symptoms, amivantamab was temporarily withheld, and topical corticosteroids were initiated, resulting in partial improvement over the following weeks. Amivantamab was subsequently resumed; however, within days of rechallenge, rapid progression to extensive scalp ulceration ensued. Attempts at self-management with over-the-counter topical products further aggravated the lesions through irritant dermatitis, and treatment with clobetasol proved insufficient, ultimately prompting hospital admission.

Physical examination revealed necrotic crusting, serosanguinous discharge, and tender ulcerations that progressed to confluent deep ulcerations involving the vertex, bilateral parietal, and occipital scalp, with significant surrounding erythema (more pronounced on the left), extensive matting of hair to the scalp, visible eroded lesions, and malodorous purulent drainage (Fig 1). Wound cultures initially grew multidrug-resistant organisms, including Providencia rettgeri, Pseudomonas aeruginosa, Serratia marcescens, and methicillin-sensitive Staphylococcus aureus. Suspecting a concern for bacterial soft tissue infection, broad-spectrum antibiotics (vancomycin, cefepime, ceftriaxone, and metronidazole) were administered. However, her ulcerative lesions continued to progress despite antimicrobial therapy, and repeat wound cultures were sterile. Dermatology and infectious disease consultations found no clinical evidence to support vasculitis or necrotizing fasciitis. Given the atypical ulcerative presentation, several alternative diagnoses were considered. Infectious etiologies remained an important consideration because of the initial purulent drainage and polymicrobial cultures; however, they became less likely in light of sterile repeat cultures and continued progression despite broad-spectrum antimicrobial therapy. Pyoderma gangrenosum was also considered, although the absence of classic violaceous undermined borders and the predominantly scalp-limited distribution made this diagnosis less probable.^9^ Autoimmune blistering disorders, including cicatricial pemphigoid, were considered less likely given the absence of mucosal involvement,^10^ while a chronic ulcerative scalp malignancy was considered less likely because of the relatively acute onset following amivantamab initiation. Taken together, the temporal relationship between amivantamab initiation and symptom onset, the progressive sterile scalp ulceration despite appropriate antimicrobial therapy, and the absence of a more likely alternative diagnosis made erosive pustular dermatosis of the scalp (EPDS) the most likely clinical diagnosis. Skin biopsy was deferred because the clinical presentation was considered sufficiently suggestive with EPDS and there were concerns that biopsy could further impair wound healing or exacerbate the extensive scalp ulceration. Given the severity of the scalp disease, treatment included both systemic and local therapies, including topical corticosteroids of escalating potency (triamcinolone 0.1%, clobetasol 0.05%, and hydrocortisone), systemic corticosteroids (methylprednisolone dose pack and prednisone during initial stabilization), oral antibiotics (cephalexin 500 mg, amoxicillin-clavulanate 500/125 mg, ciprofloxacin, and minocycline 100 mg), and topical gentamicin 0.1%.

**Fig 1:**
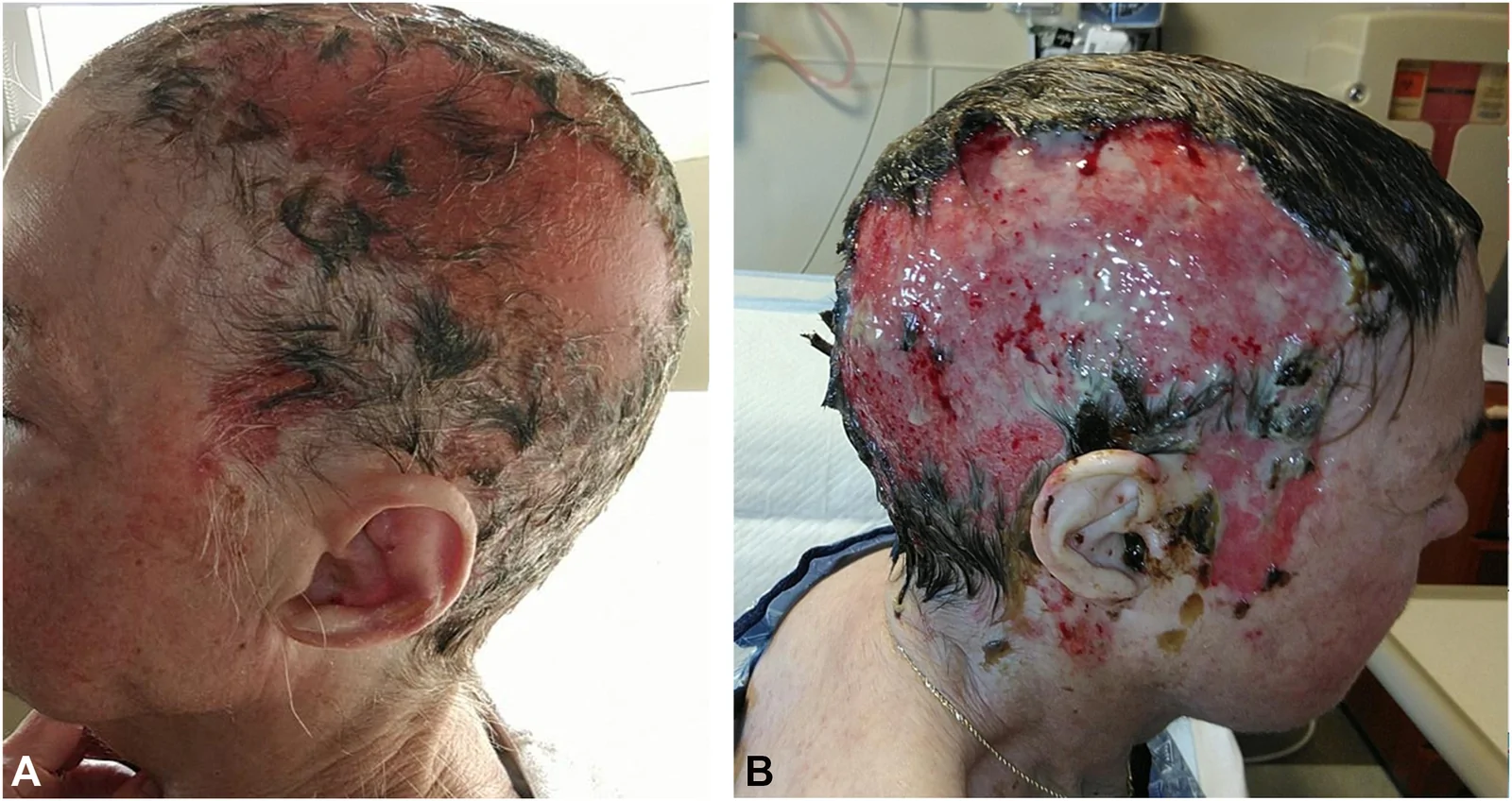
Extensive erosive and ulcerative scalp lesions involving the vertex and bilateral parietal scalp, presenting at the time of hospital admission following rapid ulceration within days of amivantamab rechallenge, and prior to initiation of topical propranolol therapy. **A,***Left side*: Partially denuded pustular dermatosis with extensive erosive and ulcerative scalp lesions involving the vertex and bilateral parietal scalp. **B,** Right lateral view showing extensive erosive and desquamating pustular dermatosis of the scalp, originating at the right temple. Copious purulent exudate and evidence of secondary infection.

Despite these interventions, clinical improvement remained minimal, and the patient was discharged with ongoing wound care. Given the refractory nature of the ulcerations despite conventional therapy, a multimodal wound-directed regimen was initiated. During the first week following discharge, an amniotic allograft (AmnioCore Pro, amnion/chorion) was applied weekly for 6 consecutive weeks because of its reported anti-inflammatory, immunomodulatory, and regenerative properties in chronic wounds. One week after allograft initiation, ultrasound-assisted debridement using an Arobella device was introduced to facilitate removal of necrotic tissue and promote a healthy wound bed and was continued for approximately 16 weeks. At approximately 10 weeks post-discharge, following completion of the allograft course, the wound developed excessive dryness, prompting transition to membrane hydrodebridement (iOLabs) to restore an appropriate moisture balance. This was continued for 2 weeks until satisfactory wound hydration was achieved. Shortly thereafter, off-label topical propranolol cream was added based on its proposed angiogenesis-modulating, anti-inflammatory, and wound-healing properties and was continued for approximately 6 weeks.^11^ Over the course of this multimodal regimen, the patient demonstrated progressive granulation tissue formation, reduction in necrotic debris and wound exudate, decreased surrounding erythema, and near-complete re-epithelialization (Fig 2). The treatment regimen was well tolerated without reported adverse effects. Amivantamab was permanently discontinued, and wound care was continued with biweekly home nursing visits for an additional 6 months.

**Fig 2:**
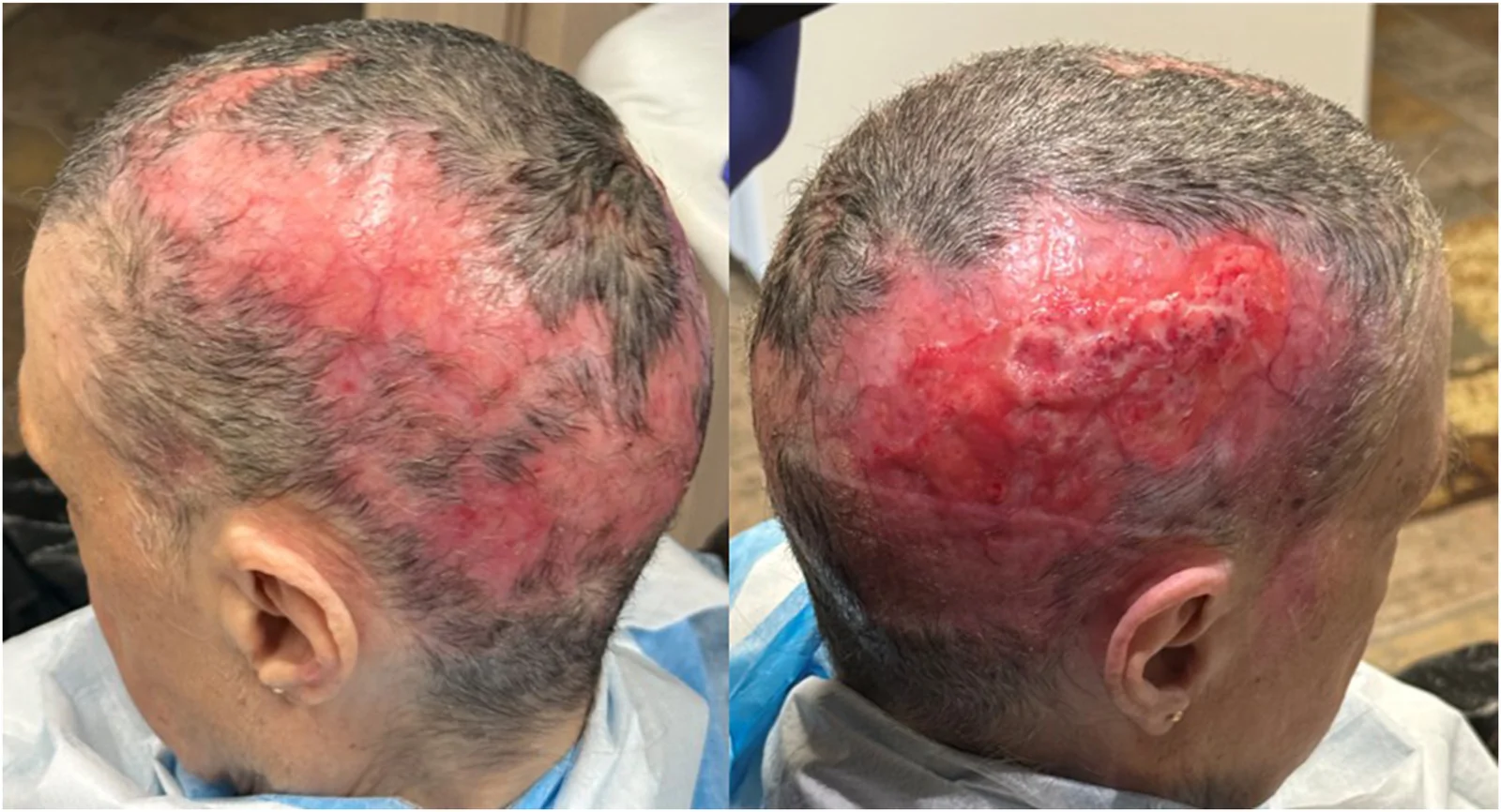
Progressive re-epithelialization and reduction in ulcer burden following 16 weeks of treatment with topical propranolol, wound debridement, and allograft therapy. Amivantamab remained discontinued during this period.

## Discussion

Amivantamab-mediated dual inhibition of EGFR and MET can disrupt epidermal homeostasis and impairs wound repair. Although scalp toxicity—including rash and ulceration—has been reported in approximately 31.1% of treated patients, EPDS remains an uncommon manifestation.^3^ Dual EGFR/MET blockade may therefore increase susceptibility to ulcerative dermatoses, particularly in individuals with preexisting actinic damage or compromised skin barrier function.^12^

EPDS is classically associated with local trauma, actinic damage, or prior interventions such as surgical procedures and skin grafting. Its nonspecific clinical presentation frequently mimics infection, malignancy, or other inflammatory dermatoses, often delaying recognition and appropriate management.^13^ Although a growing body of literature has described amivantamab-associated scalp toxicity, reports of severe, refractory EPDS-like ulceration requiring multimodal wound-directed therapy remain limited.^14^

In this case, the patient demonstrated progressive wound healing following a multimodal treatment strategy consisting of ultrasound-assisted debridement, amniotic allograft application, membrane hydrodebridement, and adjunctive topical propranolol. Amniotic allografts have been shown to provide anti-inflammatory, immunomodulatory, and regenerative effects in chronic wounds, while topical propranolol has emerged as a potential adjunct through modulation of angiogenesis and inflammation.^11^ However, the contribution of each intervention cannot be determined, and the observed improvement should be interpreted cautiously. Several confounding factors likely influenced the clinical course, including permanent discontinuation of amivantamab, treatment of the initial polymicrobial wound infection, and the patient's underlying advanced malignancies. Consequently, no causal conclusions regarding the effectiveness of any individual therapy can be drawn from this case.

In conclusion, EPDS should be considered in the differential diagnosis of persistent scalp ulcerations in patients receiving EGFR/MET-targeted therapies, particularly when lesions fail to improve despite appropriate antimicrobial treatment. Early multidisciplinary evaluation involving dermatology and wound care specialists may facilitate timely diagnosis and individualized management. Additional studies are needed to better define optimal treatment strategies for refractory amivantamab-associated scalp ulcerations and to evaluate the potential role of emerging wound-directed therapies, including amniotic allografts and topical propranolol.

## Conflicts of interest

None disclosed.
